# Temporal effects of hunting on foraging behavior of an apex predator: Do bears forego foraging when risk is high?

**DOI:** 10.1007/s00442-016-3729-8

**Published:** 2016-09-30

**Authors:** Anne G. Hertel, Andreas Zedrosser, Atle Mysterud, Ole-Gunnar Støen, Sam M. J. G. Steyaert, Jon E. Swenson

**Affiliations:** 1 Department of Ecology and Natural Resource Management, Norwegian University of Life Sciences, 1430 Ås, Norway; 2 Department of Environmental and Health Sciences, Telemark University College, 3800 Bø, Norway; 3 Institute for Wildlife Biology and Game Management, University for Natural Resources and Life Sciences, 1180 Vienna, Austria; 4 Department of Biosciences, Centre for Ecological and Evolutionary Synthesis (CEES), University of Oslo, 0316 Oslo, Norway; 5 Norwegian Institute for Nature Research, 7485 Trondheim, Norway

**Keywords:** Activity, Antipredator behavior, Bilberry, Foraging efficiency, Risk allocation

## Abstract

Avoiding predators most often entails a food cost. For the Scandinavian brown bear (*Ursus arctos*), the hunting season coincides with the period of hyperphagia. Hunting mortality risk is not uniformly distributed throughout the day, but peaks in the early morning hours. As bears must increase mass for winter survival, they should be sensitive to temporal allocation of antipredator responses to periods of highest risk. We expected bears to reduce foraging activity at the expense of food intake in the morning hours when risk was high, but not in the afternoon, when risk was low. We used fine-scale GPS-derived activity patterns during the 2 weeks before and after the onset of the annual bear hunting season. At locations of probable foraging, we assessed abundance and sugar content, of bilberry (*Vaccinium myrtillus*), the most important autumn food resource for bears in this area. Bears decreased their foraging activity in the morning hours of the hunting season. Likewise, they foraged less efficiently and on poorer quality berries in the morning. Neither of our foraging measures were affected by hunting in the afternoon foraging bout, indicating that bears did not allocate antipredator behavior to times of comparably lower risk. Bears effectively responded to variation in risk on the scale of hours. This entailed a measurable foraging cost. The additive effect of reduced foraging activity, reduced forage intake, and lower quality food may result in poorer body condition upon den entry and may ultimately reduce reproductive success.

**Electronic supplementary material:**

The online version of this article (doi:10.1007/s00442-016-3729-8) contains supplementary material, which is available to authorized users.

## Introduction

Apex predators are, by definition, exempted from interspecific predation risk in natural systems (Ordiz et al. 2013a). There is hence currently concern regarding consequences of intense human harvesting of apex predators (Darimont et al. 2015), because they do not have the same evolutionary history as prey species (Krofel et al. 2015; Ripple et al. 2014). Behavioral plasticity and alterations in response to predation risk are well-documented phenomena among prey (Lima and Dill 1990). Changes in habitat selection (Creel et al. 2005), increased vigilance (Ciuti et al. 2012b; Lima 1998), altered activity patterns (Lima and Bednekoff 1999), or changes in group size (Creel and Winnie 2005) are some of the most commonly reported antipredator responses. Most of these responses are associated with a cost, for example reduced foraging time or quality (Brown and Kotler 2004; Lima and Dill 1990). Like predation by a natural predator, human hunting has been shown to elicit antipredator behavior in hunted prey (Ciuti et al. 2012a, b; Lone et al. 2014).

A few studies have demonstrated that apex predators may perceive and respond to human-caused risk similar to that of prey responding to a natural predator (Ordiz et al. 2011), for example by shifting activity away from periods of increased human presence (Brook et al. 2012). A link between human disturbance and reduced foraging efficiency has been shown for tigers (*Panthera tigris*), which consumed less meat and abandoned carcasses more often when disturbed by humans than when undisturbed (Kerley et al. 2002). Rode et al. (2006) found that brown bears in Alaska shifted foraging activity towards the nighttime hours when bear-viewing tourists where present during the daytime hours, which they did not do in years without tourism. Further, when bears were active during the day, they were more vigilant when tourists were present, compared to when tourists were absent (Rode et al. 2006). These behavioral alterations could not be linked to decreased forage intake, however (Rode et al. 2006). Brown bears in Scandinavia avoid humans spatiotemporally by selecting for remote areas in their home ranges at times of high human activity (Martin et al. 2010). Further, bears decrease daytime activity and become more crepuscular or nocturnal in response to encounters with humans (Ordiz et al. 2013b), in areas of higher road density (Ordiz et al. 2014), or with the onset of the hunting season (Ordiz et al. 2012). Additionally, they rest in denser vegetation during daytime hours during the hunting season (Ordiz et al. 2011). Apart from intraspecific predation during the mating season (Steyaert et al. 2012; Swenson et al. 2001), humans are the sole predator on brown bears in Scandinavia and hunting is the dominant cause of bear mortalities in this population (Bischof et al. 2009). As the hunting season in Sweden coincides with the period of hyperphagia, bears should be especially sensitive to the temporal distribution of risk during this period, as they not only need to acquire sufficient energy for current survival, but also to gain fat for winter hibernation and reproduction (López-Alfaro et al. 2013). Bears in central Sweden feed almost exclusively on berries, primarily bilberry (*Vaccinium myrtillus*), during autumn (Stenset et al. 2016) and foraging brown bears select areas of higher bilberry abundance than occur randomly in the landscape (Hertel et al. 2016). Growing conditions for berries are best in open forests (Kardell and Eriksson 2011), which bears avoid during the hunting season (Ordiz et al. 2011). Further, it has been suggested that bears identify good berry patches by sight and therefore prefer to forage on berries during the daylight hours (MacHutchon et al. 1998). Bears are thus faced with the trade-off of expressing a strong antipredator behavior, at the cost of a potentially reduced forage intake or not adjusting their behavior, which potentially increases their mortality risk to hunting. According to the risk allocation hypothesis, animals adjust antipredator responses to variations in the temporal distribution, length, and intensity of predation risk (Lima and Bednekoff 1999). Animals thus should respond to short-term, high-intensity pulses of predation risk with a strong antipredator response. However, with increasing duration of risk, animals may not be able to afford a continued strong response, as they must cover nutritional demands; the behavioral antipredator responses should thus become less pronounced over time.

**Fig. 1:**
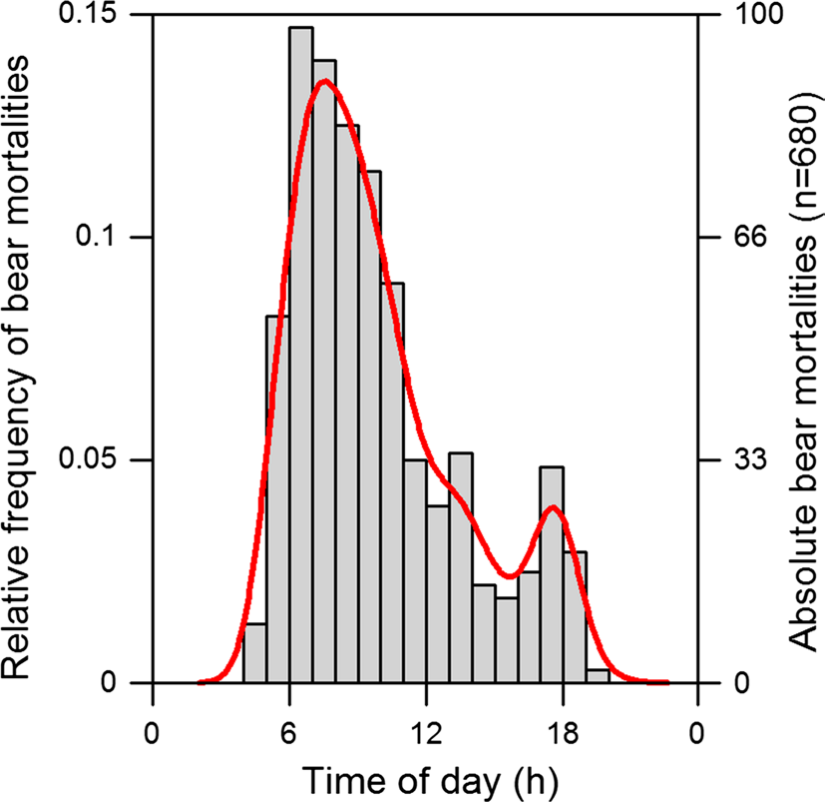
Hunting mortality risk shown as the number of brown bears shot at a given time of day during the first 2 weeks of the bear hunting season between 2006 and 2014 in and around our study area in central Sweden. Total number of shot bears = 680

Here we explore if and to what extent brown bears shift foraging activity away from high-risk time periods in the autumn during hyperphagia, as predicted from the risk allocation hypothesis (Lima and Bednekoff 1999). We consider risk intensity to primarily vary on two temporal scales over a 4-week study period, week and time of day: (a) We compared foraging activity between a 2-week period of no hunting risk (prehunting) and a 2-week period with hunting risk (hunting season). (b) On a 24-h scale, legal hunting is allowed from 1 h before the meteorological sunrise to 2 h before the meteorological sunset in Sweden (which is from 4:30 to 18:30 on 21 August, the first day of hunting). Hunting mortality risk is expected to vary throughout the day, with no risk during the night and generally higher risk in the morning hours than in the afternoon hours, due to higher hunting effort in the morning (this study, Fig. 1). Bears in our study area are generally active during the early morning hours and in the afternoon and evening, with resting periods during midday and the middle of the night (Moe et al. 2007). Based on these assumptions, we hypothesized that Scandinavian brown bears would respond to temporal variation of risk, on a scale of hours, by foregoing foraging opportunities when mortality risk is highest. Specifically, we predicted that (1) bears are less likely to forage in the hours of high risk (morning hours) during the hunting season compared to the prehunting season (no risk). We further predicted that (2) the probability that bears forage would be higher in the hours of low risk (afternoon hours) during the hunting season compared to the prehunting season (no risk), to offset potential nutritional losses from reduced foraging during the high risk hours. Days shorten at an increasingly rapid rate at these latitudes and time of the year; if bears prefer to forage during the daylight hours (Munro et al. 2006), they are therefore generally expected to increase their foraging activity during the daylight hours, i.e., forage more intensely during the shorter remaining time period (Ordiz et al. 2012). If this were the case, bears should delay foraging in the morning hours and advance foraging in the afternoon hours, essentially shortening the midday resting period, which should result in a different temporal distribution of foraging activity compared to the prehunting period. However, because the daylight hours coincide with the time of hunting, we predict (3) that bears do not adjust foraging activity towards the daylight hours, as to not increase their exposure to mortality risk, as has been suggested previously for this population (Ordiz et al. 2012). If bears do not respond to a shortening day length by delaying foraging activity into the light hours, as predicted above, this could translate into bears not finding the best patches during the darker hours. We thus predicted that, when controlling for phenological changes in berry abundance, hunter disturbance would cause bears to forage less efficiently (4) and to select berry patches of lower quality (5) in the high-risk hours, but not in the low-risk hours of the hunting season compared to the prehunting season.

## Methods

### Study area and hunting risk

The study area was situated in central Sweden, in the counties of Dalarna and Gävleborg. The area is dominated by intensively managed boreal forest. The vegetation growth period lasts 150–180 days (Moen 1998). The forest floor is dominated by berries (*V. myrtillus*, *Vaccinium vitis*-*idaea*, *Empetrum hermaphroditum*), lichens, and heather (*Calluna vulgaris*) (Swenson et al. 1999). Berries start to ripen in large quantities by the end of July and dominate the bears’ diet until den entry (Stenset et al. 2016). In our study area, bears are subject to a hunting season from 21 August until the annual quota is filled, but no later than 15 October. Harvest quotas are neither sex nor age specific, only family groups (mothers and their offspring, regardless of age) are protected from hunting (Bischof et al. 2008). Hunters are not required to apply for a permit for bear hunting and there is no limit to how many bears an individual hunter is allowed to shoot. Bears are primarily hunted with baying dogs and occasionally through still hunting during the annual moose hunt (Bischof et al. 2008). Opportunistic harvest by hunters targeting moose (*Alces alces*) has decreased in the recent years, as bear hunting has become more popular. Hunters are required to report the exact location and time of death for each bear, in addition to morphometric measurements, which are taken by an independent, state-employed examiner. Hunting regulations have been consistent during 2001–2014, but as bear population size increased, so has hunting quotas. Between 2006 and 2014, quotas doubled from 60 to 128 (for Dalarna, Jämtland and Gävleborg counties combined, Swedish National Veterinary Institute www.sva.se). We inspected time of death (rounded to the nearest full hour) for 680 bears that were legally shot during the first 2 weeks of the 2006–2014 hunting seasons (Fig. 1) to identify circadian peaks in hunting risk. In our study area, moose hunting starts on the first Monday of September. We limited our study period to the first 2 weeks of the bear hunting season as to not confuse bear- and moose-targeted hunting disturbance.

### Identifying bear foraging activity from GPS positions

We analyzed the foraging behavior of seven brown bears during the 2 weeks before (7–20 August) and 2 weeks after (21 August–3 September) the onset of the hunting season in 2014. Bears were equipped with GPS–GSM collars with a scheduled fix interval of 30 min. We downloaded positions to construct individual movement trajectories. The straight-line distance between two consecutive 30-min positions was calculated as a movement trajectory. Berry foraging was defined as slow and continuous movement behavior, in which a bear covered a distance of 25–300 m over at least three consecutive 30 min intervals (i.e. 1.5 h). Field validation confirmed that bears were foraging on berries at most of the locations [80 %, Hertel et al. (2016)]. Movement trajectories that did not concur with the distance criteria for foraging were assumed to represent other behaviors, like resting or long-distance traveling. We excluded positions with missing distance calculations and movement trajectories of less than three positions from the analysis. Based on these definitions, we identified 227 foraging trajectories, which produced 969 locations, of which we sampled 268 foraging positions, and 524 other trajectories (3970 locations). The number of identified trajectories was balanced between the pre- and posthunting seasons. We used the R package adehabitat (Calenge 2006) to construct the trajectories. We extracted sunrise, sunset, nautical dusk and dawn, and daylength for the central location of our study area (Tackåsen, Sweden: 15.05, 61.5) on every study day using the R library maptools (Bivand and Lewin-Koh 2014). Nautical dawn and dusk are defined as the time of day when the sun is between 6° and 12° below the horizon (Ensing et al. 2014). We defined twilight as the periods between the start of nautical twilight until sunrise and sunset until the end of nautical dusk, respectively.

### Field sampling of foraging and random positions

To assess the foraging efficiency of bears, we visited foraging locations from the identified foraging trajectories. To avoid autocorrelation of berry abundance at successive locations, we only sampled the second position of each trajectory. If trajectories covered more than 3.5 h (i.e., >7 consecutive positions), we also sampled the second-to-the-last position in the trajectory. Additionally, we sampled randomly generated locations in the same study area and time period. To maximize sampling effort, random locations were clustered in three representative subareas within the study area, encompassing all available habitat categories (supplemental material in Hertel et al. (2016)). To avoid observer bias in the field, we randomly selected a berry sampling location by walking 0–9 m (depending on the last number of the location’s *Y* coordinate) in a randomly assigned direction (N, E, S, W, depending on the last digit of the location’s *X* coordinate). If the selected location contained obvious sign of foraging (stripped and bent twigs and fallen berries and/or leaves), we relocated the sample plot to the opposite direction from the original GPS location. We counted all ripe berries within a 1-m^2^ sample quadrate at all foraging and random locations and determined the sugar content of a random subsample of 5 ripe bilberries. Total soluble solids (TSS) were measured as %Brix (percentage of sugar in an aqueous solution; 1 %Brix ≙ 1 g sucrose in 100 g sucrose water solution) using a digital wine refractometer MA885 (Milwaukee Instruments, Inc., Rocky Mount, NC, USA). We sampled 268 foraging positions 1 to 9 days (median 3) after a bear had been in that location. For more information on sampling procedures, see Hertel et al. (2016).

### Foraging efficiency, and quality index

We used zero-truncated negative binomial and linear regression models to describe bilberry abundance (*n* = 154) and sugar content (*n* = 110) at random locations in relation to the explanatory variables ‘habitat’ and ‘Julian date’ (described in Hertel et al. (2016)). We focused on the habitats ‘mature forest’ (average tree height >10 m) and ‘clearcuts’ (mostly ground vegetation with trees <1.3 m) in our analysis, as these have been identified as main brown bear foraging habitats with high probabilities of berry occurrence in a previous study in this study area (Hertel et al. 2016). Based on these models, we predicted the expected number of ripe berries and their sugar content in these two habitats for each day of the sampling period. Next, we calculated how efficiently the bears foraged at the 268 foraging positions sampled along the movement trajectories. Efficiency was defined as the observed number of berries at a foraging location minus the expected number of berries on this day and in the specific foraging habitat (Electronic supplementary material Fig. 1). If the resulting efficiency was >0, the foraging location contained more berries than expected from what was available; if <0, the locations contained a lower berry abundance than was available, and when it was equal to 0, the bears efficiency did not deviate from what was available. Forage quality in terms of sugar content was calculated in the same fashion (*n* = 187) (Electronic supplementary material Fig. 2).

**Fig. 2:**
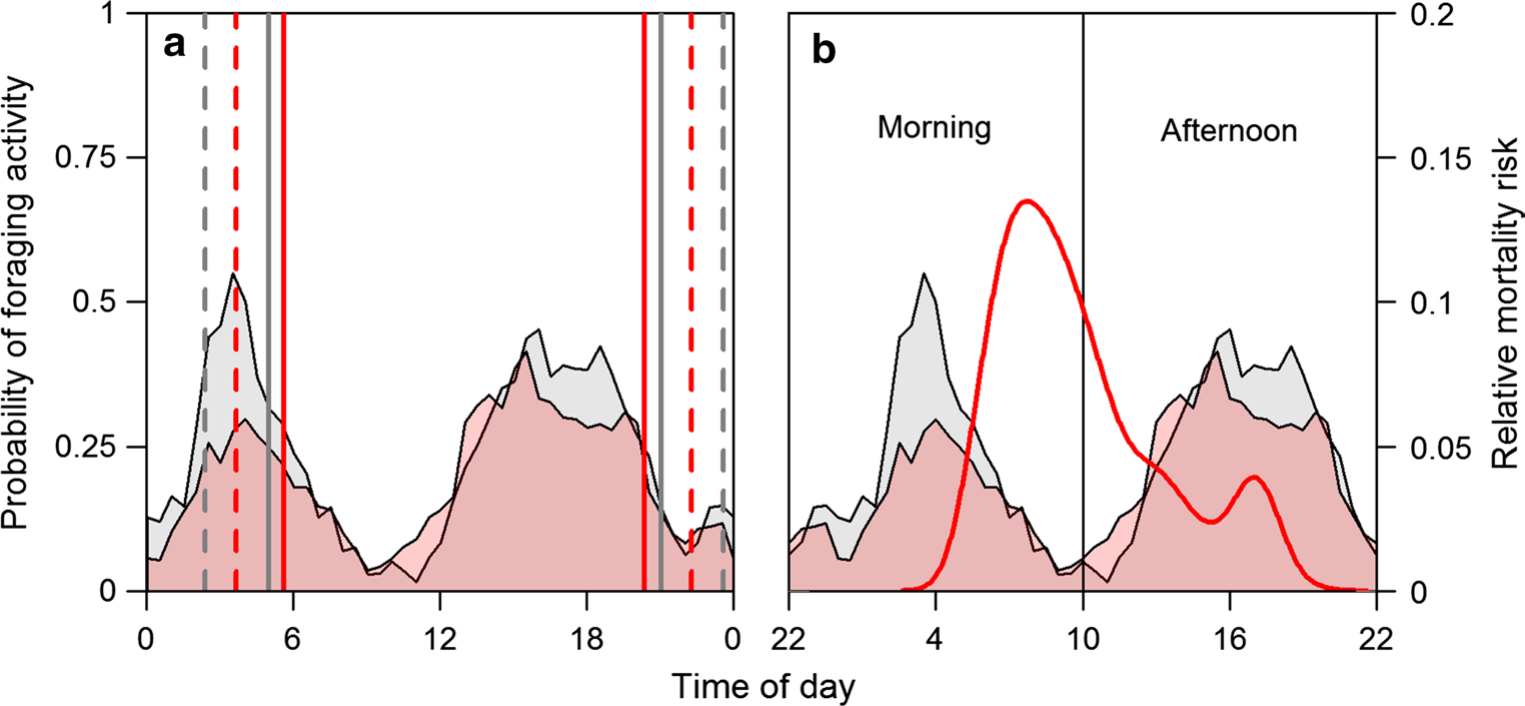
Combined foraging activity profile of seven GPS collared brown bears **a** during the 2 weeks immediately before the onset of hunting (*gray*) and first 2 weeks of the hunting season (*red*). Solid lines present mean time of sunrise and sunset, dashed lines of the mean onset of nautical twilight and end of nautical dusk. **b** Overlap of activity profiles and density of hunting risk during the day (Fig. 1). Note the rescaling of the time axis showing the partition into morning and afternoon activity adopted in all statistical analyses

### Analysis of foraging activity

We constructed a foraging activity profile of the seven bears for the 2 weeks prehunting and the first 2 weeks of the hunting season. Probability of foraging at a given time of day was calculated as the proportion of foraging fixes of the total number of all fixes, combined at this time of day for all seven individuals in either the prehunting or hunting period.

### Factors affecting foraging probability, efficiency, and diet quality

Foraging activity of bears in our study area is bimodally distributed (Moe et al. 2007). From the activity profiles, we visually determined the natural break point between the two major activity peaks and divided the day into two 12-h periods. The same break point was used in the analyses of foraging probability, efficiency, and quality. To assess whether there was a difference in these four foraging measures between the prehunting and the hunting season, we formulated a set of six candidate models (Table 1). We hypothesized that foraging efficiency was affected by the onset of the bear hunting season (prehunting vs. hunting) in the morning activity bout, but not in the afternoon activity bout. To test for a nonlinear effect of daytime on foraging, we included a cubic spline across hour of the day. We formulated models testing for time effects nested in hunting regime (prehunting vs. hunting) and for the main effect of hunting. We controlled for individual differences in performance by including bear id as a random intercept in all models. We assessed the relative support for each model using AICc and selected the model with fewest degrees of freedom (df) within an AICc range of 2 (Arnold 2010). Efficiency and quality models were implemented as generalized additive mixed models (GAMM) with normal error structure in the mgcv package (Wood 2011). Models without a time effect were fitted as generalized linear mixed models (GLMM) using the nlme package (Pinheiro et al. 2013), with a normal data distribution and method set to maximum likelihood. Because diagnostic plots of efficiency models suggested non-normality of residuals, we refitted models with square root transformed response variables. Due to negative values in the dataset, a constant was added prior to the transformation. This constant was subtracted from the predicted efficiencies after back transformation. Foraging probability was modeled as GAMM and GLMM with binomial distribution using the gamm4 (Wood and Scheipl 2013) and lme4 packages (Bates et al. 2014), respectively. We used diagnostic plots to validate that the distribution of residuals was normal and homogeneous. We were not able to fit GAMM models for the entire day because, due to bear behavior, our dataset included few data points around midday for the measures of efficiency, and quality. This led to temporal heterogeneity in the residuals. Also, additive models are sensitive to variations in sampling effort (Zuur et al. 2014). We therefore split the day into two activity periods, based on the natural break points and analyzed all measures for the two periods separately.

**Table 1:** Candidate models and hypotheses explaining foraging efficiency and selected forage quality of brown bears in Sweden

Explanatory variables	Rationale
Daytime^3^*hunt	Foraging varies over time, but in a different fashion in the prehunting and hunting periods. Additionally foraging is also affected by the hunting period itself
Daytime^3^:hunt	Foraging varies over time, but in a different fashion in the prehunting and hunting periods. Hunting period itself does not affect foraging
Daytime^3^ + hunt	Foraging varies over time in a similar fashion in the prehunting and hunting periods. Additionally, foraging is also affected by hunting period itself
Daytime^3^	Foraging is only affected by time
Hunt	Foraging is only affected by hunting period
1	Variations in foraging cannot be explained by hunting period or time of day

Models were implemented as generalized additive mixed models including a cubic spline for time of day. This term allows for a nonlinear effect on the response variable. The term hunt is a factor specifying if an observation was taken in the prehunting or hunting period. The interaction in the first two models further allows that the effect of daytime on the response variable differs between the prehunting and hunting period

We validated that our sampling method, i.e. the number of days between a bear’s visit and the sampling of a location (range 1–9, median 3) did not affect our measure of efficiency, in either the prehunting or hunting season. For both seasons we fitted a linear model with efficiency explained by number of days until sampling took place. To assess the effect, we inspected whether the confidence intervals included zero.

All statistical analyses were executed in the software R (R Development Core Team 2013).

## Results

### Foraging activity and probability

Bears showed a bimodal activity pattern during the prehunting and the hunting period (Fig. 2a). The probability of short-distance movements between positions, defined here as foraging activity, peaked between 02:00–07:00 and 13:00–20:00 and was lowest around 10:00 in both periods. This resulted in a natural split into morning and afternoon foraging activity bouts of 12 h each (Fig. 2b). The morning activity peak partly overlapped with the period of highest mortality risk, but risk peaked later than foraging probability (Fig. 2b).

**Fig. 3:**
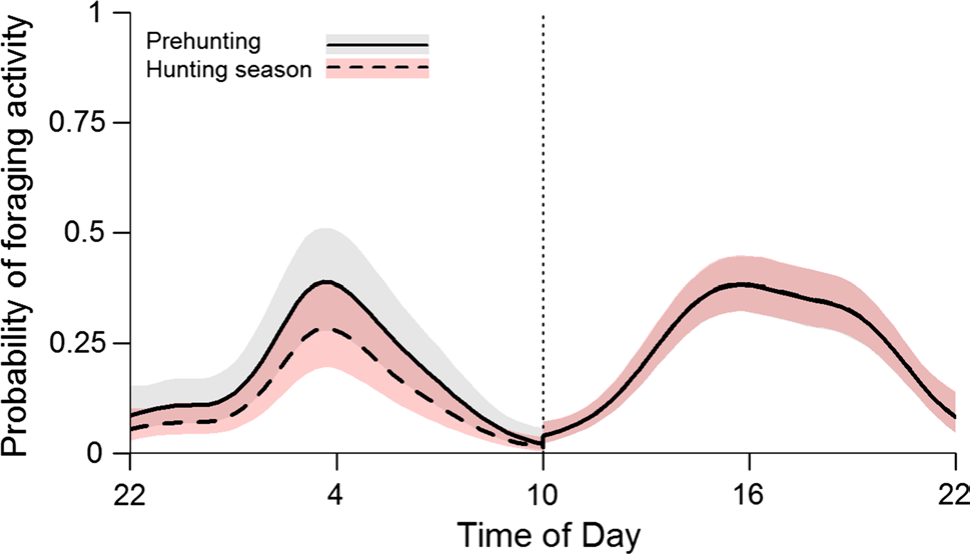
Predicted probability of foraging by brown bears during the morning (*left*) and afternoon (*right*) activity bouts. Estimates are shown as *solid lines* for the prehunting period and as *dashed lines* for the prehunting period. Polygons present 95 % confidence intervals. Estimates and CI’s for the afternoon bout are identical for the prehunting and hunting periods, as hunting did not affect foraging probability

As expected, foraging probability varied in a nonlinear fashion across time in both the morning and afternoon activity bouts (Fig. 3). Foraging probability was affected by hunting period only in the morning bout. An interaction between time of day and hunting period did not improve model fit (Table 2), which means that bears did not shift foraging to different times, but merely decreased total foraging activity. Bears were on average 10 % less probable to forage in the morning hours during the hunting period compared to the prehunting period (Fig. 3). There was no effect of hunting period on foraging probability in the afternoon activity bout, however.

**Table 2:** Performance and model selection of candidate models explaining foraging probability, efficiency, and forage quality in the morning and afternoon activity bouts of brown bears in central Sweden in the autumn 2014

Foraging probability		Morning (*n* = 2372)				Afternoon (*n* = 2550)			
	*df*	#	AICc	∆AIC	AICcw	#	AICc	∆AIC	AICcw
Daytime^3^*hunt	8	2	1878.53	6.77	0.03	4	2561.58	3.80	0.09
Daytime^3^:hunt	7	3	1882.62	10.86	0.00	3	2561.13	3.36	0.11
Daytime^3^ + hunt	6	**1**	**1871.76**	**0.00**	**0.96**	2	2559.77	2.00	0.22
Daytime^3^	5	4	1884.57	12.81	0.00	1	**2557.77**	**0.00**	**0.59**
Hunt	4	5	2019.67	147.91	0.00	5	2763.28	205.50	0.00
1	3	6	2022.23	150.47	0.00	6	2761.51	203.73	0.00

Foraging efficiency		Morning (*n* = 126)				Afternoon (*n* = 142)			
	*df*	#	AICc	∆AIC	AICcw	#	AICc	∆AIC	AICcw
Daytime^3^*hunt	8	3	594.1	2.5	0.17	5	688.42	3.82	0.06
Daytime^3^:hunt	7	6	601.1	9.54	0.01	2	686.16	1.57	0.2
Daytime^3^ + hunt	6	2	593.52	1.97	0.22	6	689.43	4.84	0.04
Daytime^3^	5	5	600.3	8.75	0.01	4	687.27	2.67	0.11
Hunt	4	**1**	**591.55**	**0.00**	**0.58**	3	686.71	2.12	0.15
1	3	4	597.91	6.35	0.03	**1**	**684.59**	**0.00**	**0.43**

Forage quality		Morning (*n* = 81)					Afternoon (*n* = 106)			
	*df*	#	AICc	∆AIC	AICcw		#	AICc	∆AIC	AICcw
Daytime^3^*hunt	8	4	253.30	7.31	0.02		6	337.37	10.60	0.00
Daytime^3^:hunt	7	6	258.51	12.52	0.00		5	335.18	8.41	0.01
Daytime^3^ + hunt	6	2	248.44	2.46	0.20		4	332.92	6.15	0.03
Daytime^3^	5	5	253.96	7.98	0.01		3	330.82	4.05	0.08
Hunt	4	**1**	**245.99**	**0.00**	**0.69**		2	328.72	1.95	0.24
1	3	3	250.26	4.28	0.08		**1**	**326.77**	**0.00**	**0.64**

Models are ranked by decreasing complexity. Best performing models within an AIC range of 2 are highlighted in bold

### Foraging efficiency

**Fig. 4:**
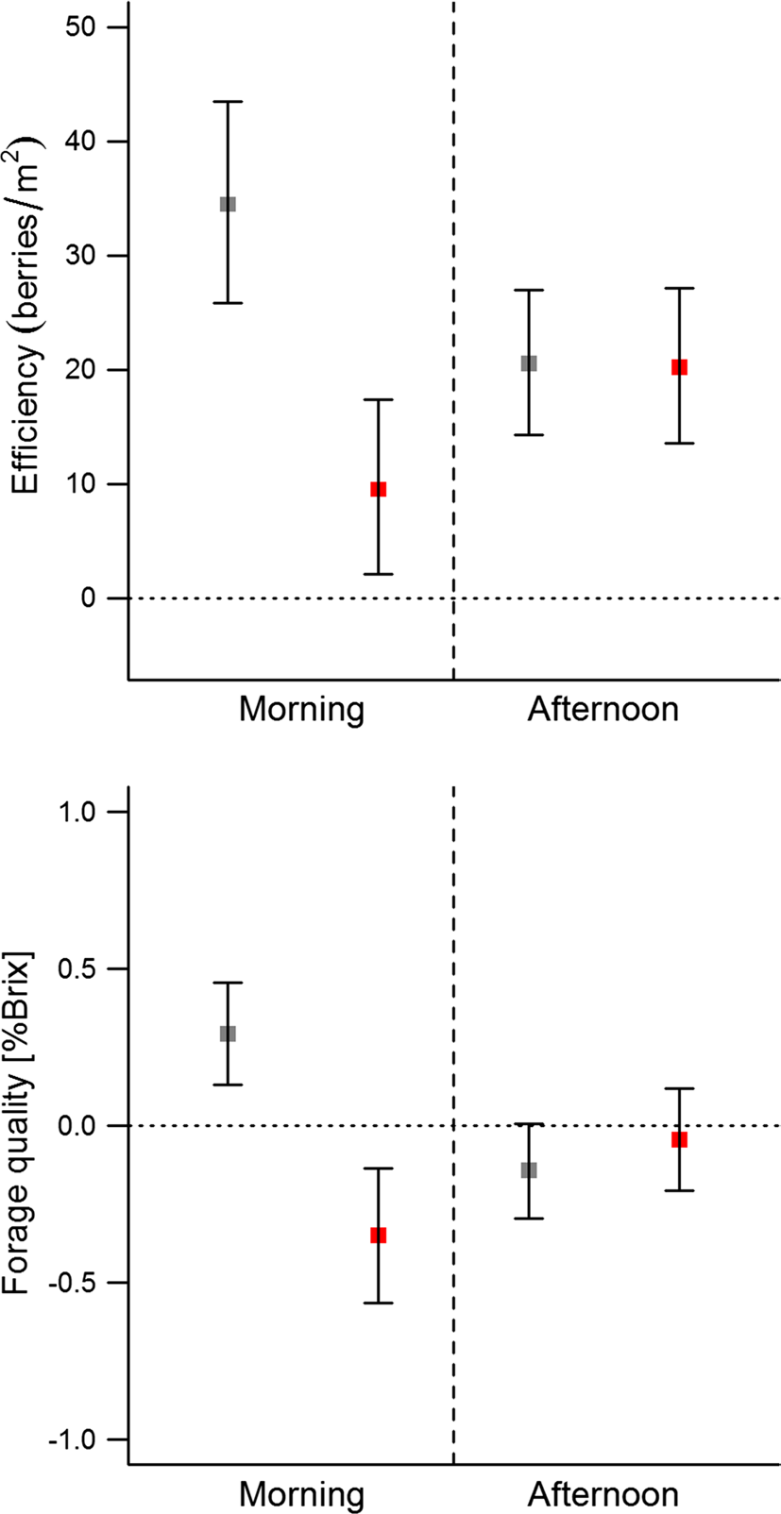
Brown bear foraging efficiency (*top*) and quality (*bottom*) during the morning and afternoon activity bouts. Estimates and standard errors are displayed for the prehunting (*gray*) and hunting (*red*) periods. Estimates represent population level means

In the morning activity bout, hunting negatively affected foraging efficiency (Tables 2, 3). During the prehunting period, bears used locations with on average 34 more berries per m^2^ than expected, whereas during the hunting period this efficiency dropped by a factor of three to only 10 berries more than expected (Fig. 4). Efficiency in the afternoon activity bout did not vary between the prehunting and hunting periods (Table 2). Bears used locations with on average 20 more berries per m^2^ than expected in both the prehunting and hunting periods, respectively (Fig. 4). There were no effects of time on foraging efficiency, in either the morning or the afternoon activity bouts. The number of days between a bear’s visit to and sampling of a location did not influence or measurement of efficiency in either the prehunting (est = –0.05, CI = –0.41 to 0.31), or the hunting season (est = –0.04, CI = –0.25 to 0.16).

**Table 3:** Coefficients and standard errors (β ± SE) for explanatory variables retained in the most parsimonious model predicting foraging probability, efficiency and quality for the morning and afternoon activity bouts of bears in relation to hunting in central Sweden

		Morning			Afternoon	
		*β*	SE		*β*	SE
Foraging probability	(Intercept)	-2.27	0.23	(Intercept)	-1.38	0.09
	Hunt (1=hunting)	-0.47	0.12			
		edf	Chi.sq		edf	Chi.sq
	Daytime^3^	5.65	143.30	Daytime^3^	5.04	169.50
		*β*	SE		*β*	SE
Foraging efficiency	(Intercept)	7.71	0.5	(Intercept)	8.39	0.31
	Hunt (1= hunting)	-1.48	0.5			
		*β*	SE		*β*	SE
Forage quality	(Intercept)	-0.35	0.22	(Intercept)	-0.09	0.12
	Hunt (1= hunting)	-0.64	0.25

### Forage quality

Forage quality in the morning activity bout was best explained by hunting period (Table 2). Bears selected berries of higher quality in the prehunting period compared to the hunting period (Table 3; Fig. 4). However, in the afternoon the intercept-only model was the best performing model, i.e., bears selected berries of similar quality in the prehunting and hunting periods. There was no temporal pattern to suggest that bears used better-quality berries at particular times of the day.

## Discussion

We found that an apex predator, the Scandinavian brown bear, responded to fine-scale temporal variation in human-caused risk due to hunting, similar to what has been observed in many prey species with an evolutionary history with predators. In line with our prediction 1, bears were less likely to forage during the most risky hours (morning) when hunting risk was present, compared to a period without hunting risk. However, the bears did not compensate for the lost foraging opportunities by increasing foraging activity at times of low risk (afternoon) during the hunting season, as compared to the prehunting season (prediction 2). Further, despite decreased daylight, bears did not adjust their foraging activity towards the daylight hours, probably to avoid risk, as we predicted (3) based on a previous study (Ordiz et al. 2012). Importantly, this had negative effects on their foraging efficiency. Bears foraged less efficiently, in terms of food abundance (number of berries/m^2^, prediction 4) and food quality (nutritional value of berries, prediction 5), during the risky morning hours in the hunting period compared to the prehunting period. Our findings support the risk allocation hypothesis, that animals should elicit strong antipredator responses during short, high-intensity risk pulses (Lima and Bednekoff 1999). This highlights that the same predictive framework developed for prey can be used for apex predators exposed to human harvesting.

Empirical support for a flexible response of prey to the immediate presence of predators, in relation to the general risk setting (i.e., the proportion of time under high predation risk), comes from the Yellowstone elk-wolf (*Cervus canadensis, Canis lupus*) system (Creel et al. 2008). There elk that are under regular predation risk from wolves are less vigilant than elk that are only occasionally exposed to wolves. Although seemingly counterintuitive, vigilance is costly in terms of foraging (Christianson and Creel 2010; Winnie and Creel 2007) and elk populations at constant risk might not be able to afford continuous strong antipredator responses.

How risk avoidance can cause foraging costs due to (a) feeding time allocation and (b) spatial variability of risk (Brown and Kotler 2004) has been demonstrated, e.g. for elk (Ciuti et al. 2012b) and red deer (*C. elaphus*) (Lone et al. 2015). Elk in Canada showed highest levels of vigilance on public lands, where there were more hunters, during the hunting season and increased levels of vigilance explained 40 % of variation in feeding time (Ciuti et al. 2012b). Surviving male red deer in Norway selected denser habitats in periods of high hunting risk compared to periods of low hunting risk, and these habitats had 68 % less coverage of bilberry plants, an important forage for red deer (Lone et al. 2015).

Brown bears show great behavioral plasticity throughout their distribution range and within populations. North American bears are generally more day active than European bears (Kaczensky et al. 2006), which has been attributed to a shorter persecution history (Zedrosser et al. 2011). Nevertheless, undisturbed bears in Canada and Alaska forage more during daylight hours, whereas disturbed bears shift activity towards nighttime hours with low human disturbance (MacHutchon et al. 1998; Rode et al. 2007) and this has been suggested to reduce berry forage intake, because bears may use vision for food search (MacHutchon et al. 1998). Animal prey, such as salmon (*Oncorhynchus* spp.), may in fact be less vigilant during twilight or dark hours, which might be beneficial for fishing bears, outweighing the costs of reduced visibility and may result in equal or higher catch rates during dark compared to light hours (Klinka and Reimchen 2002, 2009). Further, human disturbance can cause spatial displacement of foraging bears from a food resource, when alternative food resources away from human presence are available (Rode et al. 2007).

We compared measures of foraging activity and efficiency within the same population of brown bears on two time scales, a 2-week period of predator absence (prehunting) and a 2-week period of predator presence (hunting season), and, nested therein, in daily periods of low (afternoon) and high predation risk (morning). Circadian variations in risk levels proved important in predicting the bears’ allocation of antipredator behaviors, which was ultimately associated with lower foraging efficiency. The proximate mechanism may be that bears stay in denser vegetation after disturbances (Ordiz et al. 2011), where berry shrubs produce fewer berries with lower sugar content (Kardell and Eriksson 2011). Bears also reduce their movements in risky situations (Ordiz et al. 2012), which may result in reduced accessibility to the best berry foraging locations. Elk change their diet as a consequence of antipredator behavior (Christianson and Creel 2008, 2010). However, as previously shown, bears did not change their use of food resources during the 4-week study period (Hertel et al. 2016). Also, growing conditions for lingonberry (*V. vitis*-*idaea*), the only other available food resource, are best on open clearcuts, a habitat that is expected to be avoided by bears when risk is high (Ordiz et al. 2011). It is therefore unlikely that our results were confounded by bears using other food resources than bilberry more often during the hunting season. In addition, bears did not offset reduced forage intake by foraging for longer time spans when risk was low. During the less risky afternoon hours, bears did not increase their foraging activity or efficiency of food intake in the hunting period compared to prehunting.

Bears reduced their foraging activity in the morning hours, even though bear activity peaked earlier than hunting risk. The reduction in activity corresponded in time to the actual times when bears were killed, clearly suggesting that this was a response to elevated risk during the opening of the bear hunting season. As most bears are hunted with dogs (Bischof et al. 2008), time of kill is often preceded by a prolonged pursuit. Hunting disturbance is therefore not limited to the time of shooting, but to a generally increased activity level in the landscape, which includes people driving into the forest, pursuit through the forest, and transporting shot animals out of the forest. Dogs are also more likely to pick up a fresh track of a bear that has been active recently than an older track. This might decrease the detection probability for bears that decrease their activity during the morning activity bout even before hunters arrive in the forest.

Ordiz et al. (2013b) have shown that bears alter their behavior for up to 3 days after a close encounter with a human. However, one conclusion of our study is that the actual risk of being shot and a bear’s perception of risk coincide, even though bears might not encounter a human directly. This can be explained by a general increase of human and dog activity in the forest, because the start of the bear hunting season coincides with the first day that all dogs are allowed to be off-leash in the forest after a 6-month leash requirement. Many hunters other than bear hunters use this opportunity to train their dogs. Further, traffic on forest roads increases, presenting a direct disturbance, but also facilitating human access to remote areas. Bears in our study area respond to this by becoming more nocturnal in areas of higher road density (Ordiz et al. 2014).

Our results clearly showed that bears altered their foraging patterns in response to the onset of the hunting season and that this was associated with a foraging cost. However, only animals in good condition can afford to forgo foraging in favor of an antipredator response (Lima and Bednekoff 1999). In bears, cubs of the year, subadults (<4 years), and pregnant females should be the demographic groups that must increase their body mass the most to ensure survival [young bears: Schwartz and Franzmann (1992)] and to gain extra weight for reproduction [pregnant females: Elowe and Dodge (1989); López-Alfaro et al. (2013)]. If bears show a long-lasting behavioral response to hunting disturbance, reduced forage intake over a prolonged time could translate into reduced reproductive output if foraging is sufficiently reduced to affect body condition (Christianson and Creel 2008, 2010; Creel et al. 2011; Lima 1998). However, our results suggest that bears are able to detect variations in human disturbance, and thereby mortality risk, on a temporal scale of hours and their behavioral response may therefore be rather immediate and short term. These findings are supported by experimentally disturbed bears, which decreased foraging activity during the immediate time of disturbance (Ordiz et al. 2013b; Rode et al. 2007). Managers may want to consider further limiting the time of day during which bear hunting is allowed to minimize the adverse effects on food intake during the crucial period of hyperphagia.

## Electronic supplementary material

Below is the link to the electronic supplementary material.

Supplementary material 1 (PDF 205 kb)
