# Supplementary material for: Temporal effects of hunting on foraging behavior of an apex predator: Do bears forego foraging when risk is high?
Source: Oecologia. 2016 Sep 30;182(4):1019–29. doi: 10.1007/s00442-016-3729-8 (PMC5061844; doi:10.1007/s00442-016-3729-8)

Electronic supplementary material

Temporal effects of hunting on foraging behaviour of an apex predator: Do bears forego foraging when risk is high?

A. G. Hertel, A. Zedrosser[b], A. Mysterud, O.-G. Støen, S. M. J. G Steyaert, J. E. Swenson

Fig. 1. Observed number of bilberries at brown bear foraging locations (dots) on clearcuts and in mature forest in central Sweden. Samplings took place between 7 August and 3 September. Lines represent predicted number of berries in a given habitat based on samplings of random points. Foraging efficiency at each foraging locations was calculated as the number of berries found at the foraging location (dots) minus the expected number of berries on this day/habitat (line).

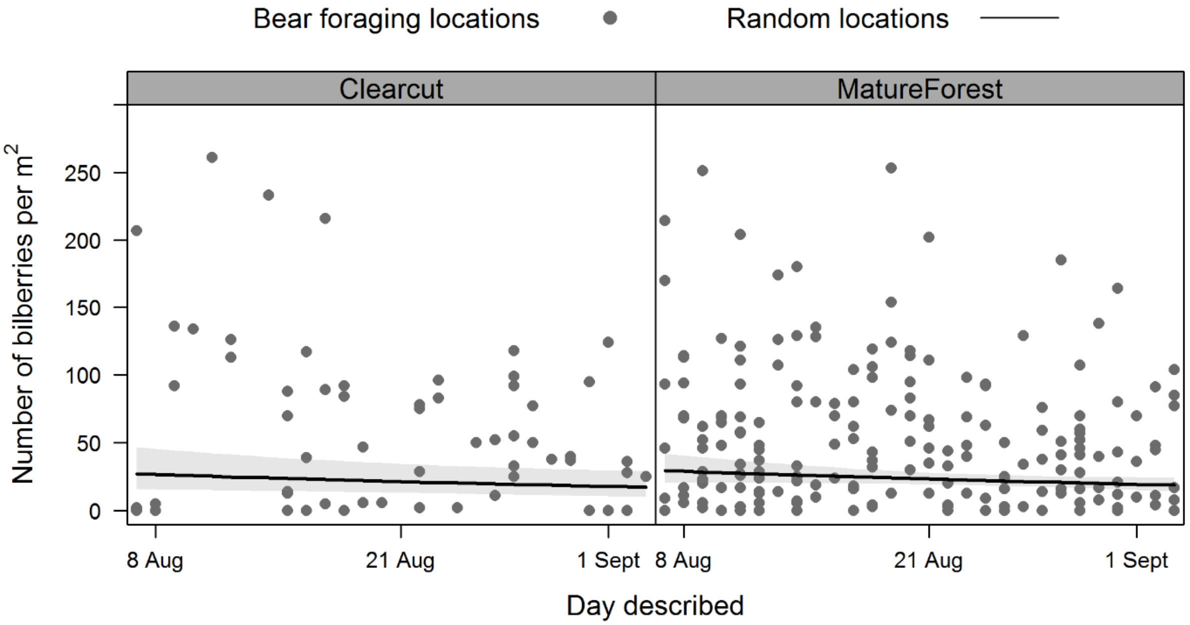

Fig. 2. Illustration of forage quality calculation based on sugar content in bilberries for brown bears in two habitats in central Sweden during 7 August and 3 September. Foraging efficiency at each sampled locations was calculated as the residual of sugar content of berries found at the foraging location minus the expected sugar content of berries on this day/habitat (based on random samplings in the landscape).

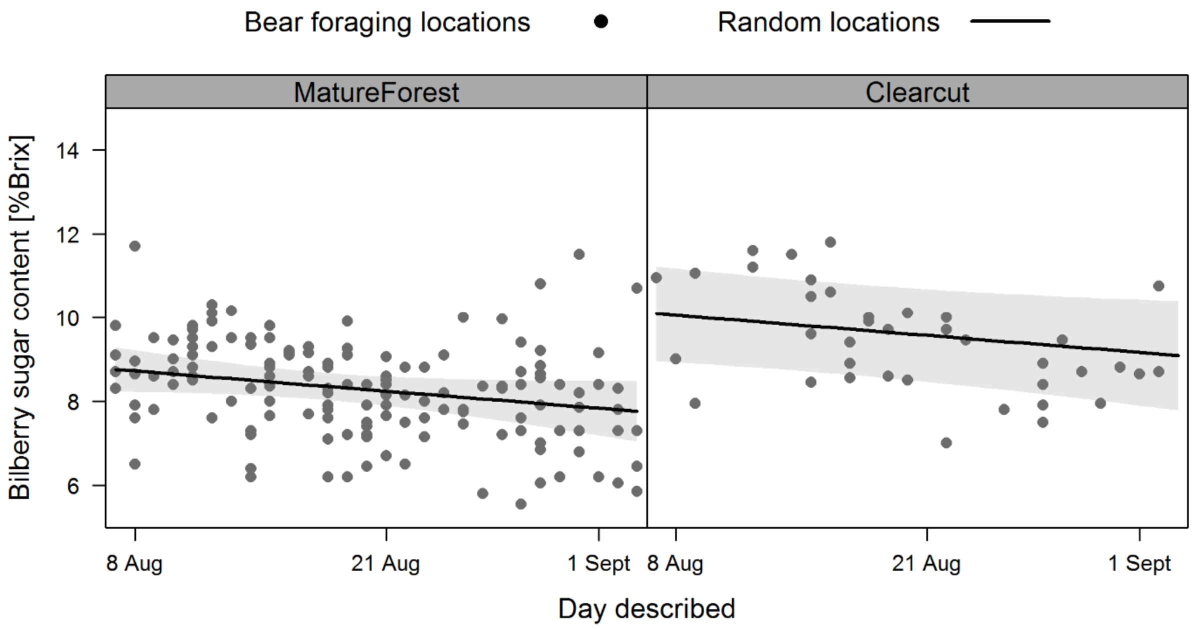

Supplement: Supplementary file 1 — Supplementary material 1 (PDF 205 kb) [file 442_2016_3729_MOESM1_ESM.pdf]
